# Supplementary material for: Reciprocal impacts of telomerase activity and ADRN/MES differentiation state in neuroblastoma tumor biology
Source: Commun Biol. 2021 Nov 19;4:1315. doi: 10.1038/s42003-021-02821-8 (PMC8604896; doi:10.1038/s42003-021-02821-8)

Reciprocal impacts of telomerase activity and tumor cell differentiation in neuroblastoma tumor biology

Eun Young Yu, Syed Zahid, Sarah Aloe, Erik Falck-Pedersen, Kathy Zhou, Nai-Kong V. Cheung, and Neal F. Lue

Supplementary Figures 1-9

Supplementary Table 1-2

Supplementary Figure 10: Original Gel and Blot images

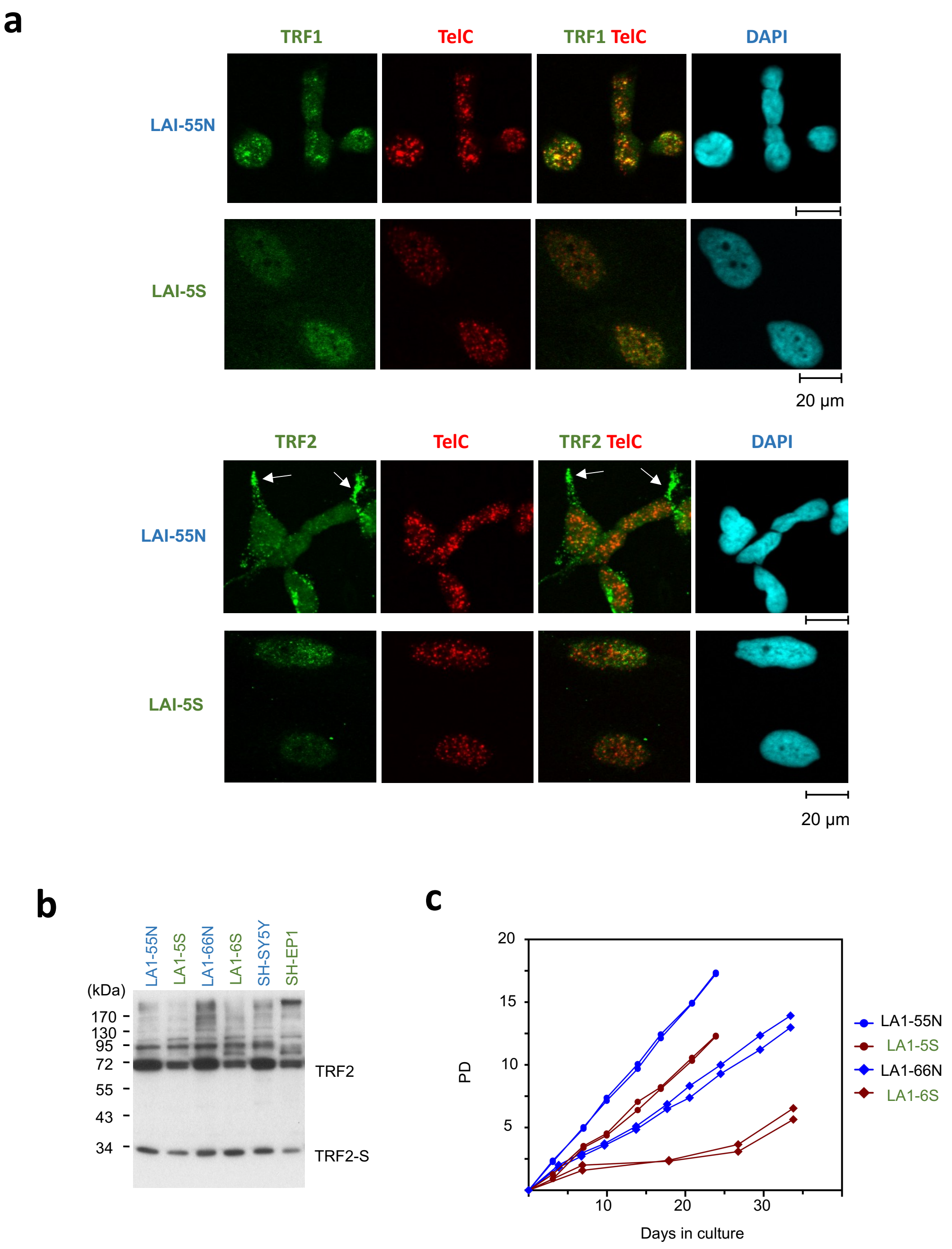

Supplementary. Fig. 1. Differential cellular distribution of telomere protein in matched ADRN and MES cell lines

(a) and (b) IF-FISH analysis of TRF1 and TRF2 in the indicated cell lines. Prominent examples of peri-nuclear staining of TRF2 in LA1-55N are marked white arrows.

(b) Western analysis of TRF2 and TRF2-S. TRF2-S was detected by longer exposures of TRF2 Western blots.

(c) Comparison of growth rate in two matched ADRN and MES cell lines. The LA1-55N (ADRN) and LA1-5S (MES) pair and LA1-66N (ADRN) and LA1-6S (MES) pair were used.

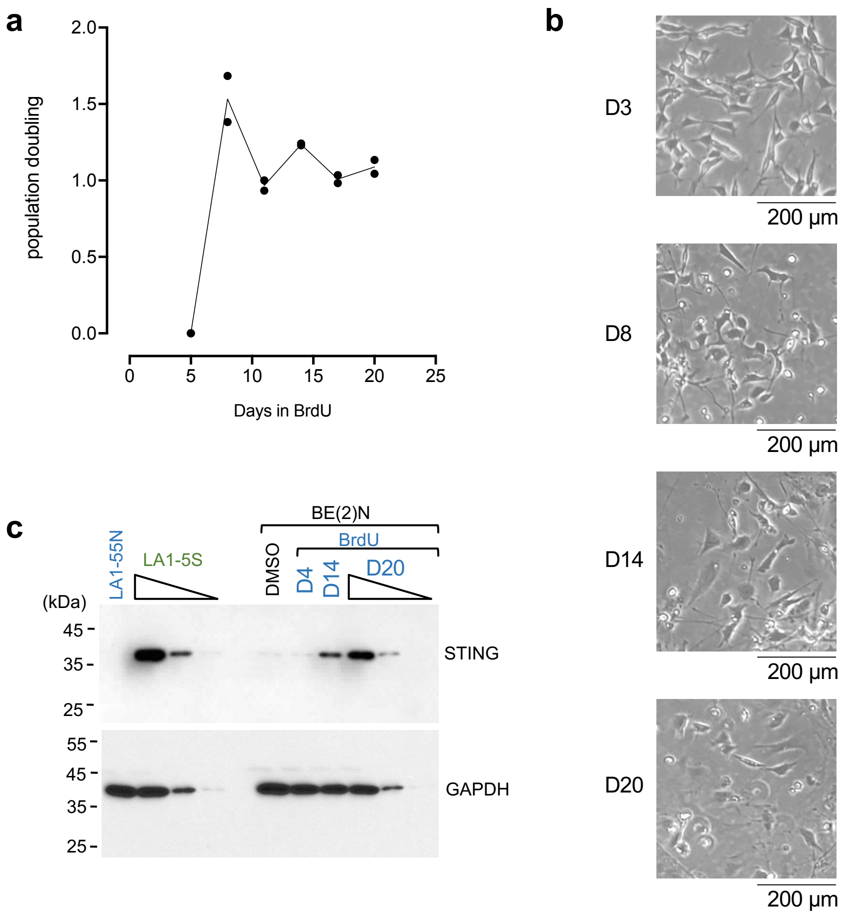

Supplementary Fig. 2. Characterization of BE(2)N proliferation and morphology during BrdU-induced switch from ADRN to MES cells

(a) BE(2)N cells were treated with 7.5 µM BrdU. On day 4, cells were split and seeded into multiple 100 mm dishes at equal densities, followed by further incubation in 7.5 µM BrdU. Cell numbers in the dishes were determined at regular intervals from day 4 to day 20, converted to population doubling, and plotted.

(b) Representative images of BE(2)N cells on day 3, 8, day 14, and day 20 in the cell counting experiment are displayed.

(c) Western analysis of STING in a pair of matched ADRN and MES cell lines (LA1-55N and LA1-5S), and in BE(2)N during BrdU treatment. Five µg of each sample were analyzed. Serial 3-fold dilutions of LA1-5S and BE(2)N at Day 20 were used to estimate the quantitative difference in protein levels.

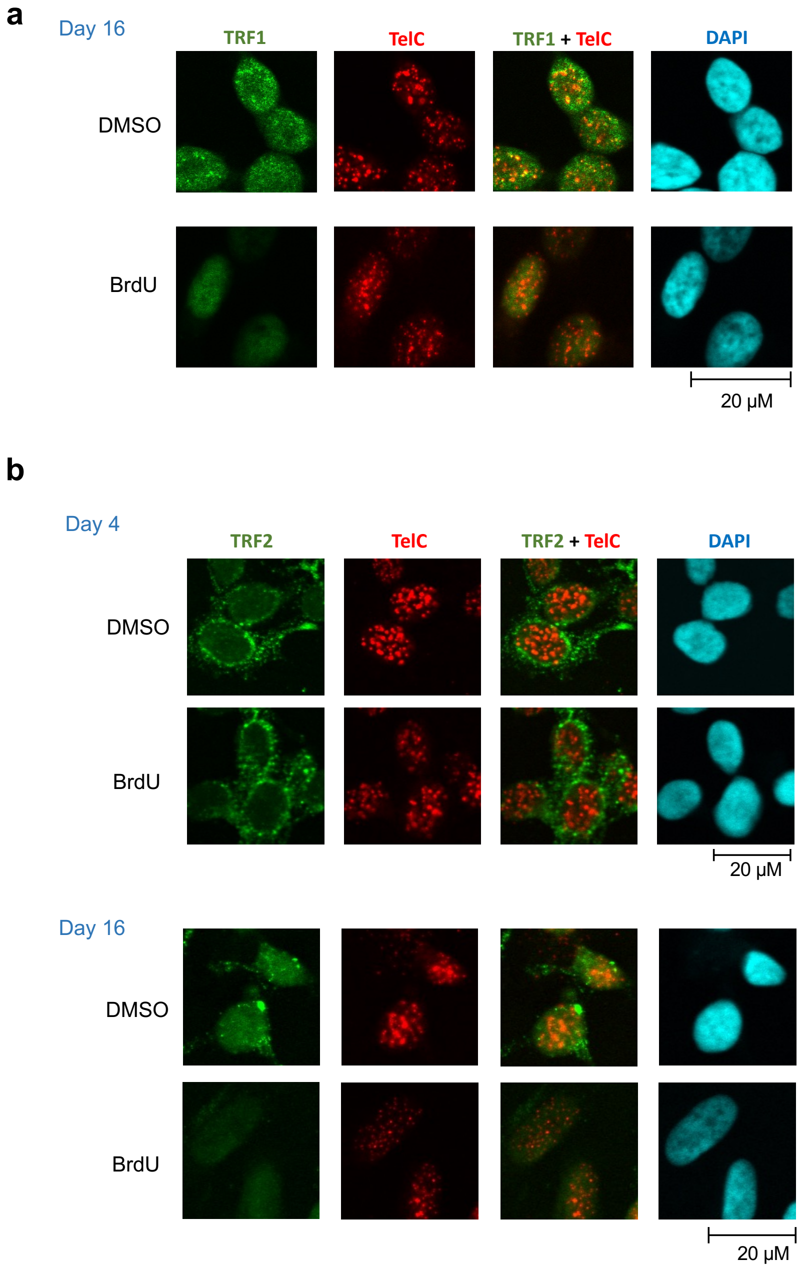

Supplementary Fig. 3. Cellular distribution of TRF1 and TRF2 in BE(2)N cells during BrdU-induced switch from ADRN to MES cell types

(a) and (b) IF-FISH analysis of TRF1 and TRF2 in BE(2)N at different time points during BrdU-induced phenotypic switch.

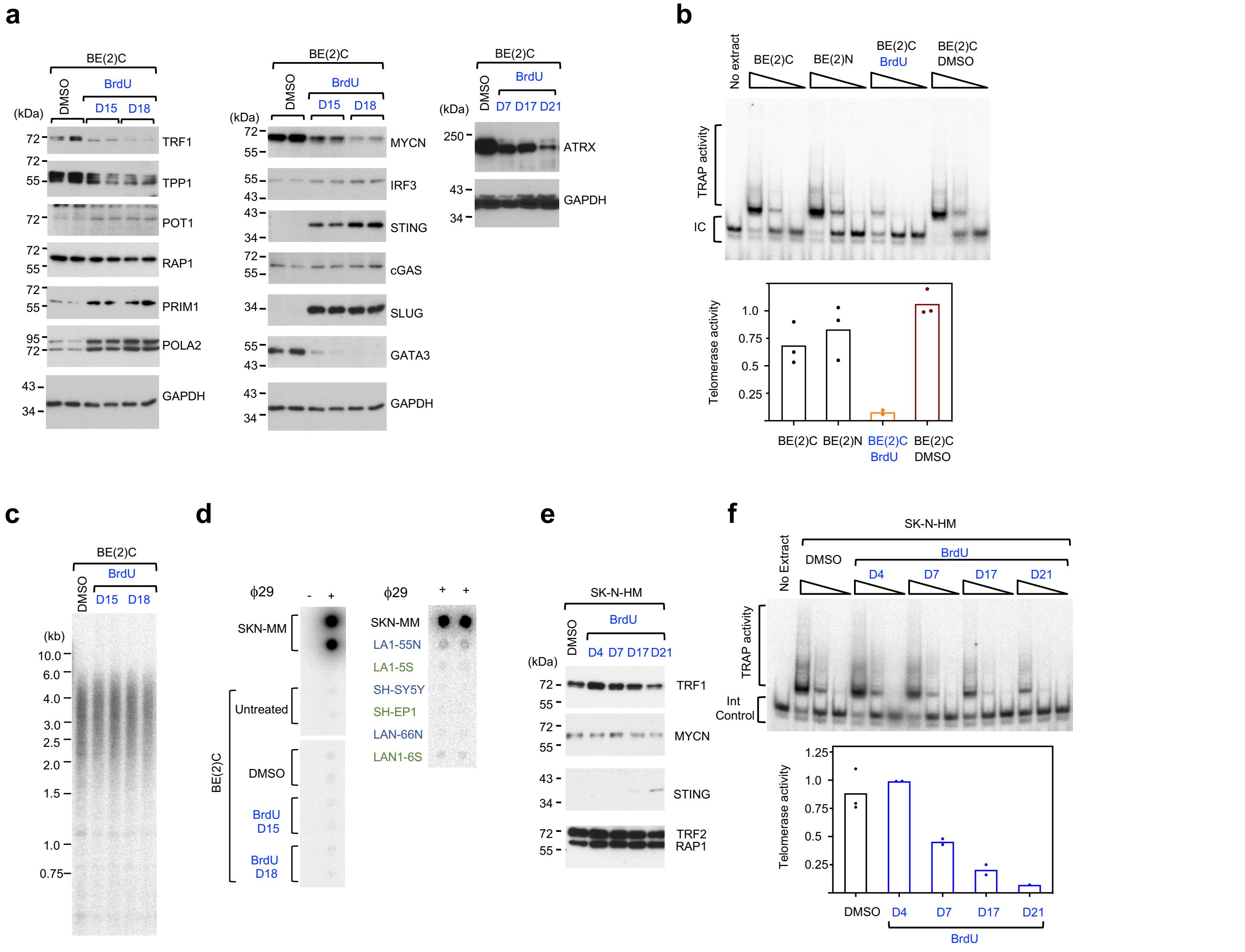

Supplementary Fig. 4. Profiling of BE(2)C and SK-N-HM cells during BrdU-induced switch from ADRN to MES cells

(a) Western analysis of telomere-, lineage- and immunity-related factors in BE(2)C during BrdU-induced phenotypic switch.

(b) TRAP analysis of telomerase activity in BE(2)C at different time points during BrdU-induced phenotypic switch. The assays were performed using serial dilutions of the extracts (equivalent to 500, 50 and 5 ng of proteins), and the relative activities are plotted at the bottom (means, n=2 or 3 experimental replicates).

(c) Analysis of telomere length distributions in BE(2)C at different time points during BrdU-induced phenotypic switch.

(d) (Left) C-circle analysis of BE(2)C in control and BrdU-treated cells. (Right) C-circle analysis of three pairs of matched ADRN and MES cell lines. For comparison, the C-circle level of SKN-MM, an ALT-positive NB cell line, was also examined.

(e) Western analysis of telomere and lineage-related and DNA sensing factors in SK-N-HM during BrdU-induced phenotypic switch.

(f) TRAP analysis of telomerase activity in SK-N-HM at different time points during BrdU-induced phenotypic switch. The assays were performed using serial dilutions of the extracts (equivalent to 500, 50 and 5 ng of proteins), and the relative activities are plotted (means, n=2 or 3 experimental replicates).

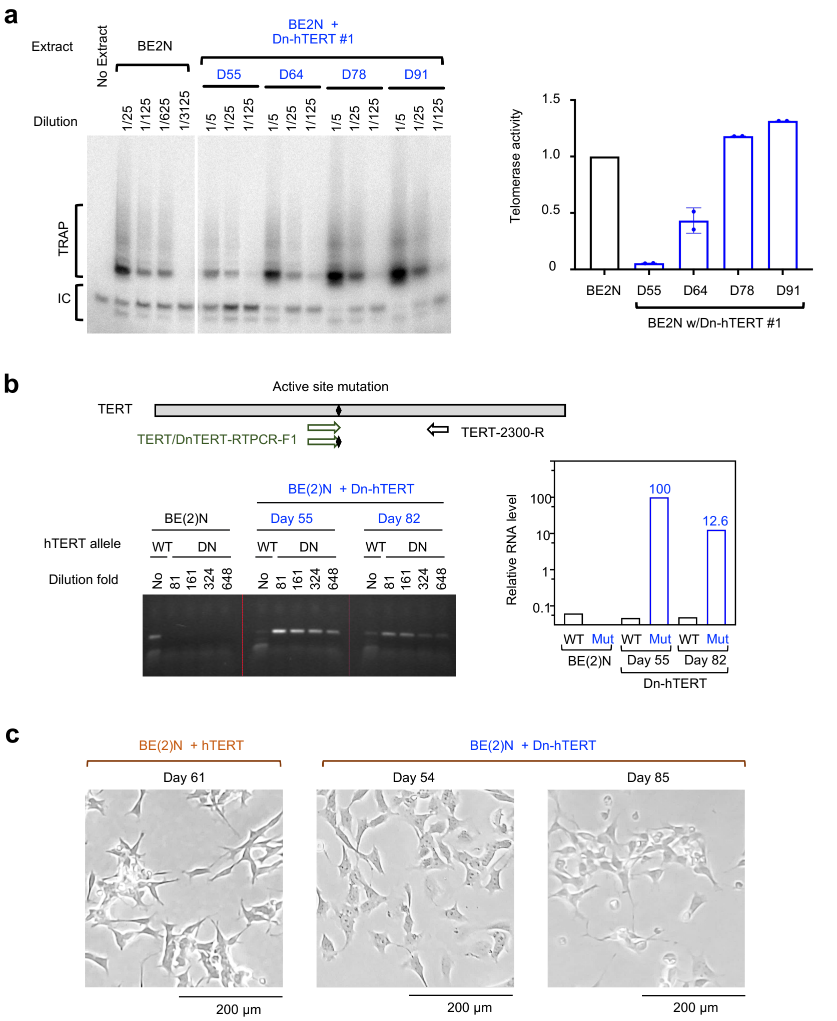

Supplementary Fig. 5. Characterization of BE(2)N cells during Dn-hTERT-induced switch from ADRN to MES cells

(a) TRAP analysis of telomerase activity in BE(2)N harboring either hTERT or Dn-hTERT at different time points following retrovirus infection. The assays were performed using serial dilutions of the extracts as indicated. The relative activity of each extract was quantified using ImageQuant and plotted (mean ± S.D., n=2 experimental replicates).

(b) Allele-specific RT-PCR analysis with total RNA from parental BE(2)N and BE(2)N harboring Dn-hTERT at different time points following retrovirus infection. Total RNAs were serially diluted as indicated and applied to the assays. The relative levels of wild type hTERT and DN-hTERT mRNA were quantified using ImageQuant and plotted. The samples were from Dn-hTERT #2 cultures.

(c) Morphology of BE(2)N harboring either hTERT or Dn-hTERT (#2 culture) at different time points following retrovirus infection.

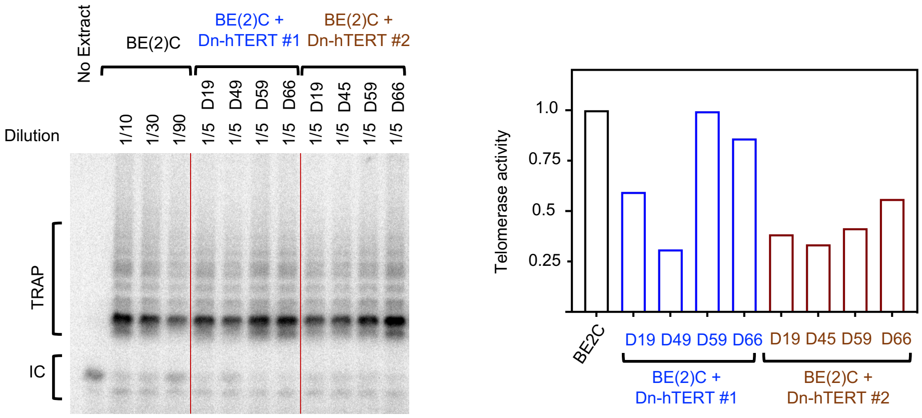

Supplementary Fig. 6. Mild inhibition of telomerase activity in Dn-hTERT treated BE(2)C

TRAP analysis of telomerase activity in BE(2)C harboring Dn-hTERT at different time points following retrovirus infection. The assays were performed using different dilutions of the extracts as indicated. The relative activity of each extract was quantified using ImageQuant and plotted.

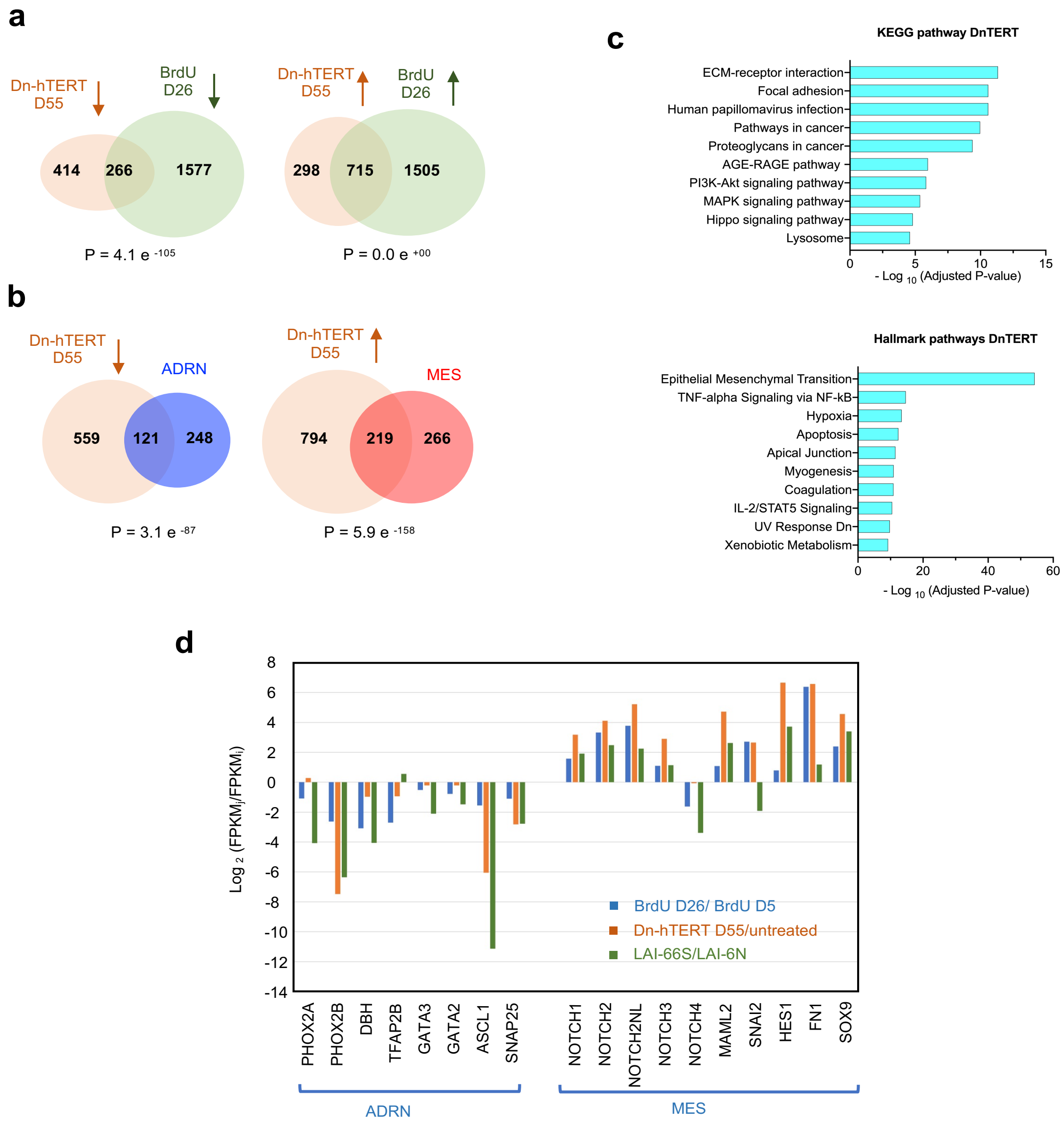

Supplementary Fig. 7. Comparison of gene expression pattern among Dn-hTERT-induced MES-like BE(2)N cells and BrdU-induced MES-like BE(2)N cells, and with previously defined MES and ADRN signature genes

(a) Pair-wise comparison of the degree of overlaps in down-regulated genes (left) and up-regulated genes (right) between Dn-hTERT-induced MES-like BE(2)N cells (day 55) and BrdU-induced MES cells (day 26).

(b) (left) The list of genes that were down-regulated in Dn-hTERT-induced MES-like BE((2)N cells (day 55) (by >2-fold) was compared to the ADRN signature list [10]. (right) In parallel, the list of genes that were up-regulated in Dn-hTERT-induced MES-like BE(2)N cells (day 55) (by >2-fold) was compared to the MES signature.

(c) The ~1,000 genes up-regulated in Dn-hTERT-treated BE(2)N (by >2-fold) were subjected to pathway analysis by the Enrichr program. The top ten GO terms identified by the KEGG pathway database and the Hallmark gene sets in the Molecular Signature Database were ranked by adjusted P-values and plotted in the top and bottom panels, respectively.

(d) The expression changes of a subset of ADRN and MES signature genes in the BrdU experiment (day 26 vs day 5) and Dn-hTERT experiment (day 55 vs parental cell) are plotted. Also included are the relative expression of these genes between a matched pair of ADRN and MES cells (LA1-66N and LA1-6S).

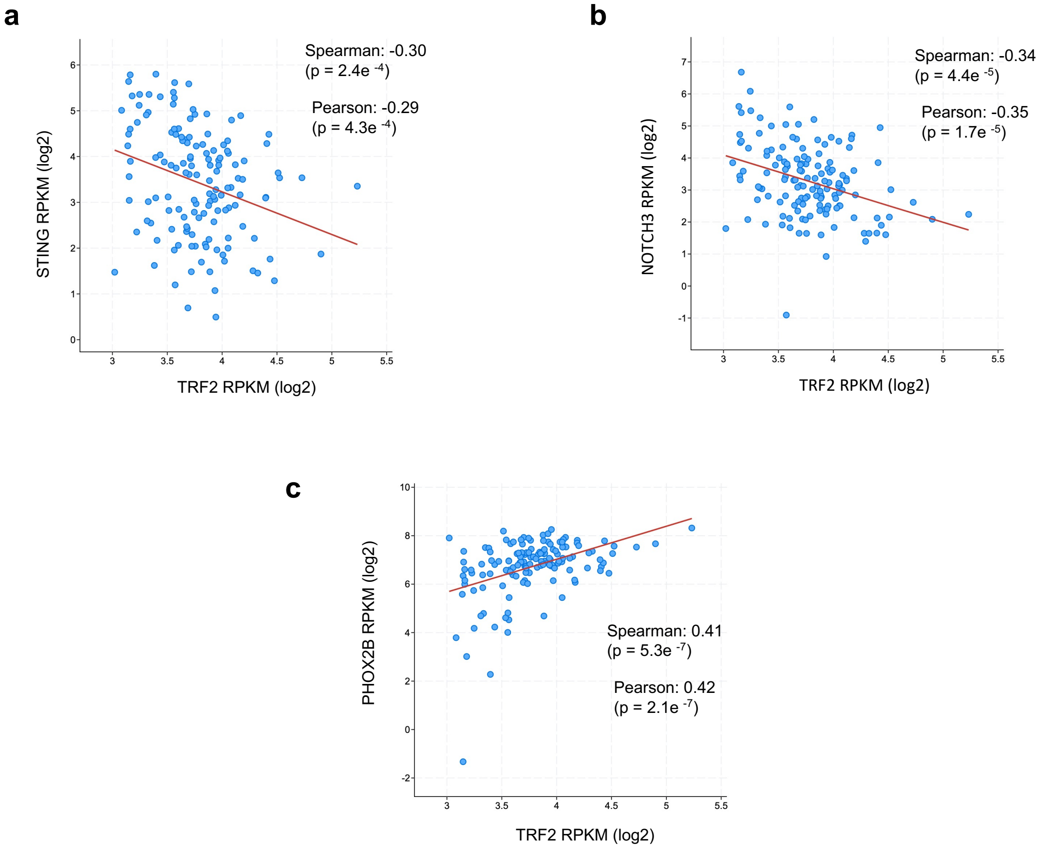

Supplementary Fig. 8. Correlations between the levels of TRF2 RNA and three other genes implicated in NB cell lineage regulation in patient samples

The RNA levels of TRF2 in relation to three genes implicated NB cell lineage determination (i.e., STING [immunity], NOTCH3 [MES marker] and PHOX2B [ADRN marker]) in NB tumors from the Pediatric Neuroblastoma (TARGET, 2018) collection were analyzed and visualized using cBioportal.

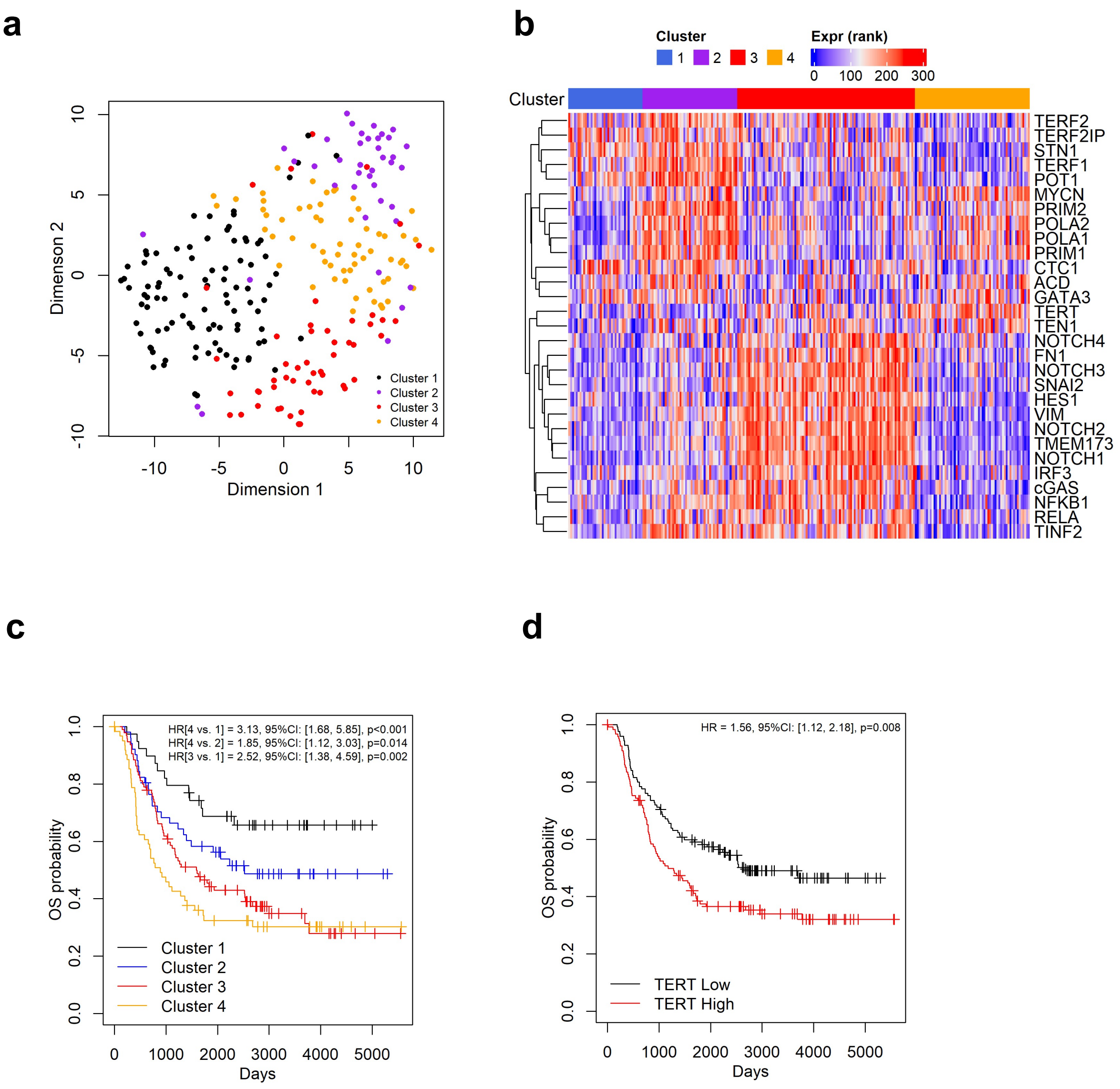

Supplementary Fig. 9. Analysis of the expression patterns and clinical outcomes associated with NB tumor samples with microarray data using a telomere- and cell lineage-related signature gene list

(a) The RNA levels (rank transformed) of a list of 29 telomere-, cell lineage- and immunity-related genes across 247 NB tumors with microarray data were analyzed and clustered using PAM and displayed as a t-SNE plot.

(b) The rank transformed RNA levels of signature genes across four clusters of NB tumor samples are displayed in Heatmap.

(c) Kaplan-Meier overall survival curves for the four clusters of patients with distinct gene expression profiles.

(d) Kaplan-Meier overall survival curves for patients in the microarray dataset with high TERT and low TERT expression.

Supplementary Table 1. Gene expression differences between cluster 1 and cluster 3 patients

	All(n=85)	1(n=35)	3(n=50)	P-value*
**AGE**				
Mean+/-sd	3.92+/-2.52	2.91 +/- 2.29	4.62 +/- 2.46	0.002
Median (IQR)	3 (2, 5)	2 (1,4)	4 (3,6)	<0.001
**INSS_STAGE, n (%)**				
Stage 2b	1(1.18%)	1 (2.86%)	0 (0%)	
Stage 3	4(4.71%)	3 (8.57%)	1 (2%)	
Stage 4	68(80%)	20 (57.14%)	48 (96%)	
Stage 4s	12(14.12%)	11 (31.43%)	1 (2%)	<0.001
**TUMOR_SAMPLE_HISTOLOGY, n (%)**				
Favorable	15(17.65%)	12 (34.29%)	3 (6%)	
Unfavorable	66(77.65%)	21 (60%)	45 (90%)	
Unknown	4(4.71%)	2 (5.71%)	2 (4%)	0.001
**RISK_GROUP, n (%)**				
High Risk	69(81.18%)	20 (57.14%)	49 (98%)	
Intermediate Risk	9(10.59%)	8 (22.86%)	1 (2%)	
Low Risk	7(8.24%)	7 (20%)	0 (0%)	<0.001
**OS_STATUS, n (%)**				
0:LIVING	37(43.53%)	21 (60%)	16 (32%)	
1:DECEASED	48(56.47%)	14 (40%)	34 (68%)	0.015
**OS_DAYS**				
Mean+/-sd	1557.44+/-1043.28	1826.94 +/- 1162.02	1368.78 +/- 916.92	0.056
Median (IQR)	1549 (659, 2325)	2064 (806,2500)	1323.5 (487.25,2012.75)	0.04
**OS_MONTHS**				
Mean+/-sd	51.68+/-34.28	60.46 +/- 38.14	45.54 +/- 30.19	0.058
Median (IQR)	51 (22, 77)	68 (27,82.5)	44 (16.75,67)	0.042
**TERT**				
Mean+/-sd	0.71+/-0.94	0.36 +/- 0.59	0.95 +/- 1.05	0.002
Median (IQR)	0.08 (0, 1.25)	0 (0,0.78)	0.65 (0.03,1.77)	<0.001
**TERF2**				
Mean+/-sd	4.01+/-0.34	3.9 +/- 0.3	4.1 +/- 0.35	0.005
Median (IQR)	3.98 (3.81, 4.15)	3.91 (3.75,4.04)	4.05 (3.86,4.27)	0.006
**TERF2IP**				
Mean+/-sd	6.59+/-0.56	6.78 +/- 0.46	6.46 +/- 0.59	0.007
Median (IQR)	6.65 (6.32, 6.94)	6.85 (6.52,7.04)	6.55 (6.05,6.72)	0.004
**POLA1**				
Mean+/-sd	2.55+/-0.56	2.19 +/- 0.52	2.79 +/- 0.43	<0.001
Median (IQR)	2.58 (2.25, 2.91)	2.26 (1.92,2.5)	2.73 (2.54,3.04)	<0.001
**POLA2**				
Mean+/-sd	3.94+/-0.92	3.45 +/- 1.02	4.29 +/- 0.67	<0.001
Median (IQR)	4.06 (3.5, 4.59)	3.53 (2.69,4.21)	4.23 (3.91,4.73)	<0.001

**STN1**				
Mean+/-sd	2.93+/-0.44	3.15 +/- 0.5	2.77 +/- 0.32	<0.001
Median (IQR)	2.89 (2.65, 3.22)	3.23 (2.9,3.42)	2.79 (2.65,2.96)	<0.001
**TEN1**				
Mean+/-sd	3.11+/-0.51	3.34 +/- 0.47	2.94 +/- 0.48	<0.001
Median (IQR)	3.14 (2.72, 3.4)	3.35 (3.14,3.55)	2.95 (2.65,3.26)	<0.001

* P-values were determined using the ANOVA and the non-parametric Kruskal-Wallis tests.

Supplementary Table 2. Antibodies and Oligos used in this study

Antibodies	Sources	Identifier	Concentration
mouse anti-TRF1	SCBT	sc-56807	1: 3,000
mouse anti-TRF2	Novusbio	NB100-56506	1: 3,000
mouse anti-RAP1	SCBT	sc-53434	1: 3,000
mouse anti-TIN2	Novusbio	NB600-1522	1: 2,000
rabbit anti-TPP1	Bethyl	A303-069A-T	1: 2,000
rabbit anti-POT1	Novusbio	NB500-176	1: 3,000
mouse anti-GAPDH	ABclonal	AC002	1: 100,000
mouse anti-TERT	SCBT	sc-393013	1: 1,000
mouse anti-STN1/OBFC1	SCBT	sc-374178	1: 2,000
mouse anti-PRIM1	SCBT	sc-390265	1: 3,000
mouse anti-POLA2	SCBT	sc-398255	1: 3,000
mouse anti-RPA70	SCBT	sc-48425	1: 1,500
rabbit anti-TZAP	Proteintech	24665-1-AP	1: 2,000
mouse anti-ATRX	SCBT	sc-55584	1: 3,000
mouse anti-RTEL1	SCBT	sc-515427	1: 3,000
mouse anti-RIF1	SCBT	sc-515573	1: 3,000
mouse anti-MYCN	SCBT, sc-53993	sc-53993	1: 3,000
mouse anti-IRF3	SCBT	sc-33641	1: 3,000
rabbit anti-STING	Cell Signaling	#13647	1: 3,000
mouse anti-cGAS	SCBT	sc-515777	1; 3,000
mouse anti-SLUG	SCBT	sc-166476	1: 2,000
mouse anti-GATA3	SCBT	sc-269	1: 2,000
rabbit anti-phospho-STAT1	Cell Signaling,	#9167	1: 3,000
rabbit anti-phospho-TBK1	Cell Signaling,	#5384	1: 3,000
HRP-linked anti-rabbit IgG	Cell Signaling,	#7074	1: 5,000
HRP-linked Anti-Mouse IgG	Cell Signaling,	#7076	1: 5,000

Oligos	Sequences
For in-gel hybridization	
TTAGGG$_4$ (G4)	TTAGGG TTAGGG TTAGGG TTAGGG
CCCTAA$_4$ (C4)	CCCTAA CCCTAA CCCTAA CCCTAA
For C-circle hybridization	
TTAGGG$_8$ (G8)	TTAGGG TTAGGG TTAGGG TTAGGG TTAGGG TTAGGG TTAGGG TTAGGG
CCCTAA$_8$ (C8)	CCCTAA CCCTAA CCCTAA CCCTAA CCCTAA CCCTAA CCCTAA CCCTAA
For TRAP assays	
TS	AAT CCG TCG AGC AGA GTT
ACX	GCG CGG CTT ACC CTT ACC CTT ACC CTA ACC
NT	ATC GCT TCT CGG CCT TTT
TSNT	AAT CCG TCG AGC AGA GTT AAA AGG CCG AGA AGC GAT

For STELA	
C Telorette 1	GCTCCGTGCATCTGGCATCCCCTAAC
C Telorette 2	GCTCCGTGCATCTGGCATCTAACCCT
C Telorette 3	GCTCCGTGCATCTGGCATCCCTAACC
C Telorette 4	GCTCCGTGCATCTGGCATCCTAACCC
C Telorette 5	GCTCCGTGCATCTGGCATCAACCCTA
C Telorette 6	GCTCCGTGCATCTGGCATCACCCTAA
Teltail	GCTCCGTGCATCTGGCATC
XpYpE2	GTTGTCTCAGGGTCCTAGTG
XpYpB2	TCTGAAAGTGGACC(A/T)ATCAG
For hTERT and Dn-hTERT RT-PCR	
DnTERT-RTPCR-F1	G TAC TTT GTC AAG GTC GCG A
TERT-RTPCR-F1	G TAC TTT GTC AAG GTG GAT G
TERT-2300-R	GTC AAG GTA GAG ACG TGG CT

Supplementary Figure 10: Original Gel and Blot images

Original images for Fig. 1a

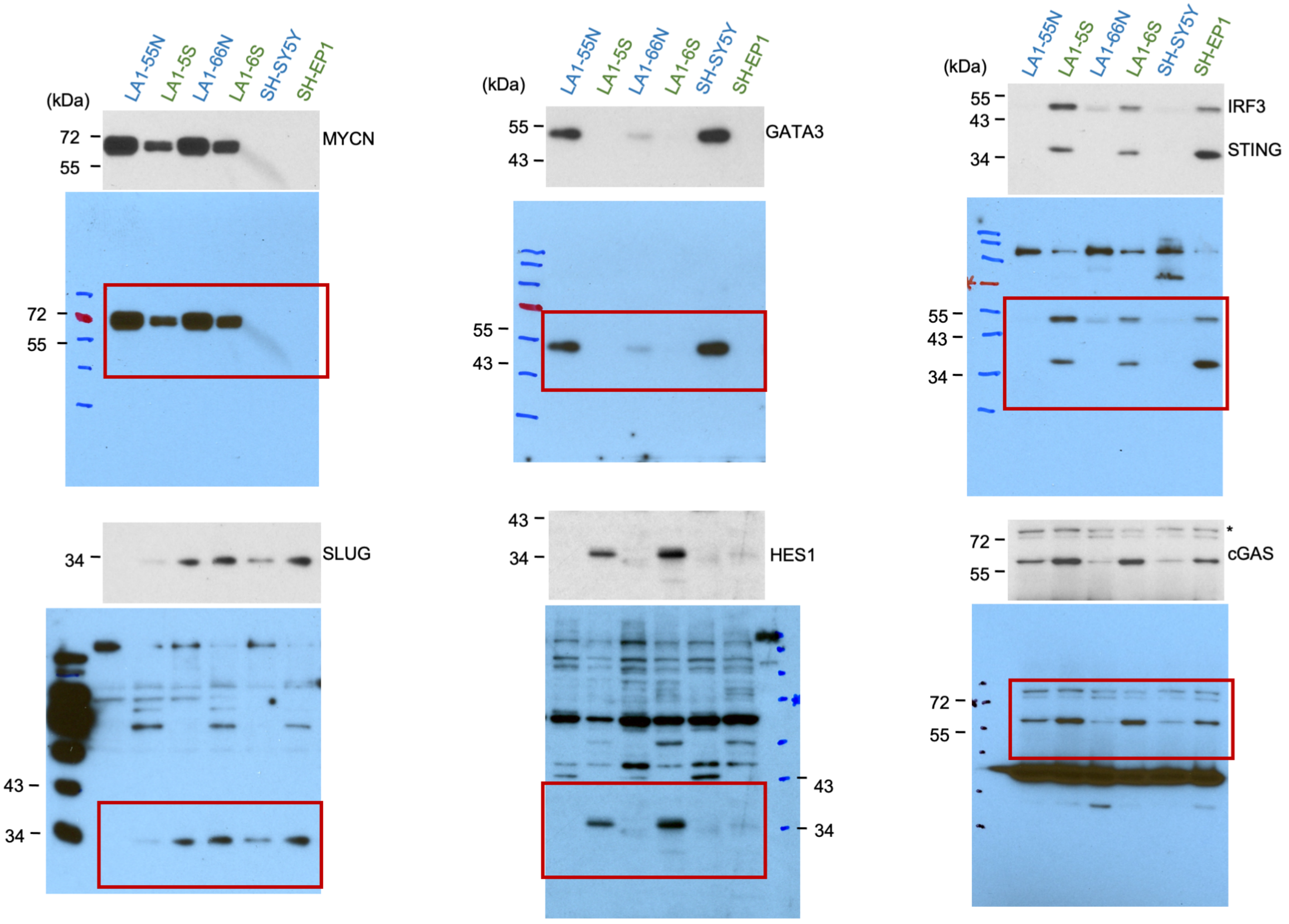

Original images for Fig. 1b (part 1)

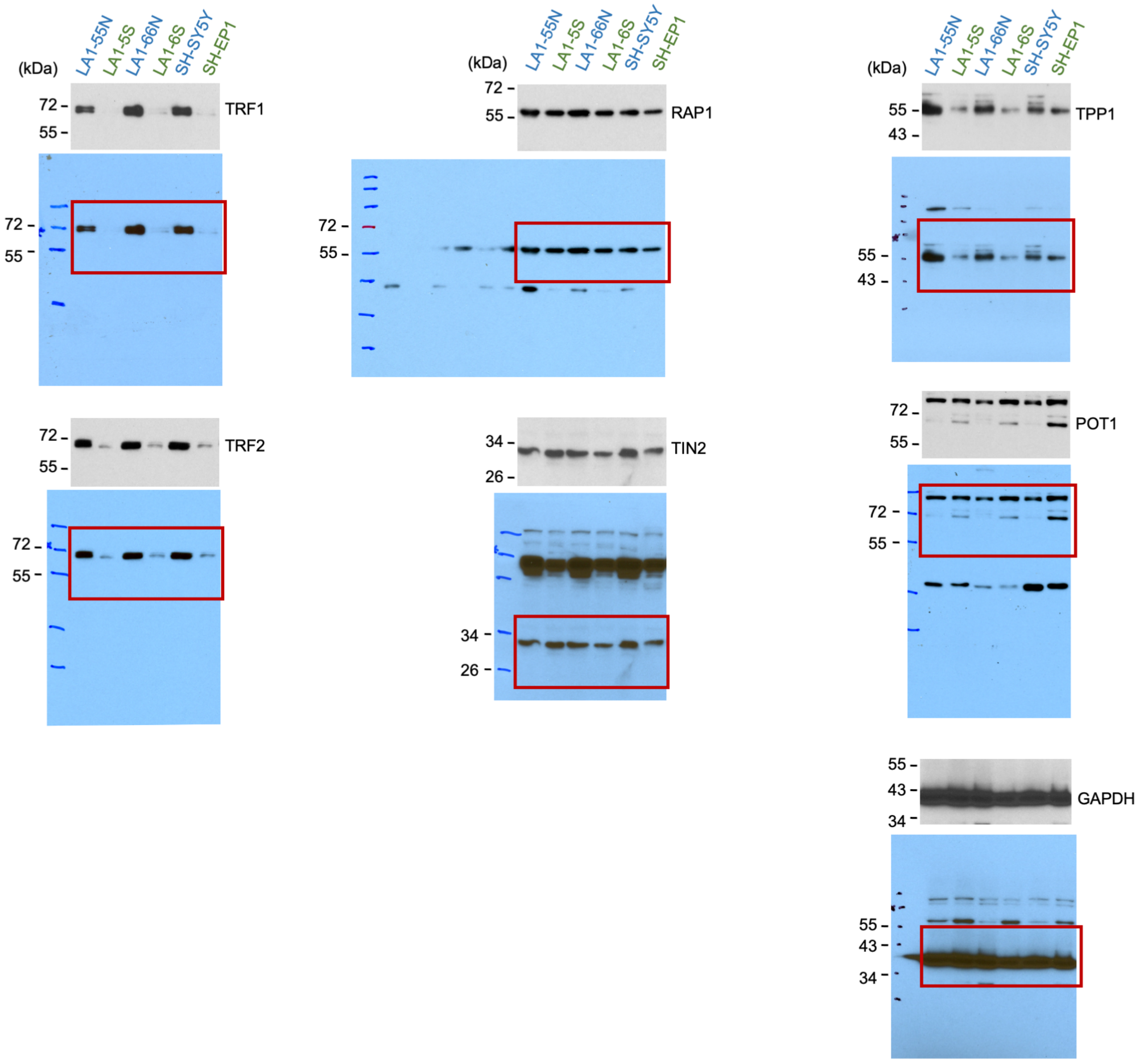

Original images for Fig. 1b (part 2)

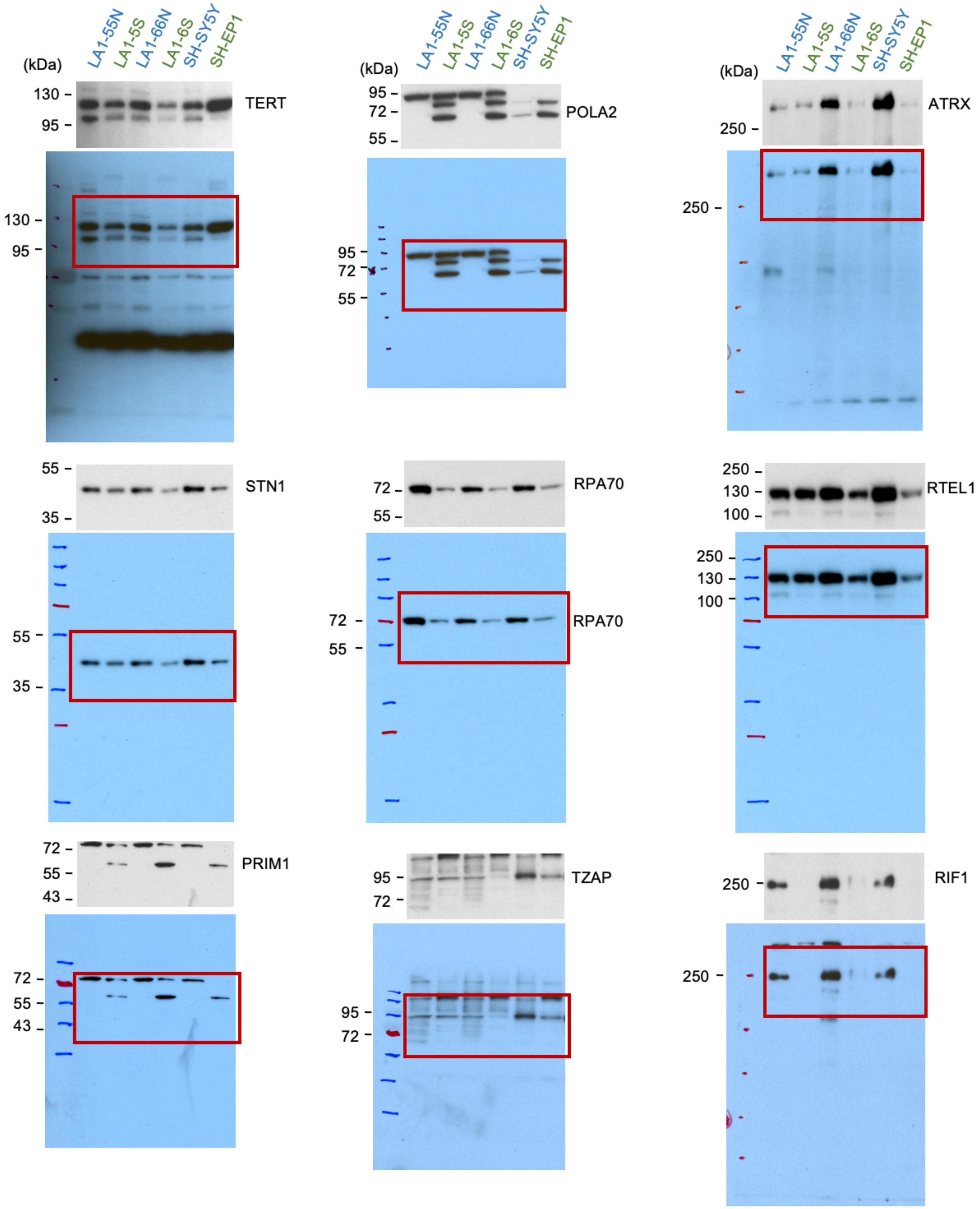

Original images for Fig. 2a and 2b

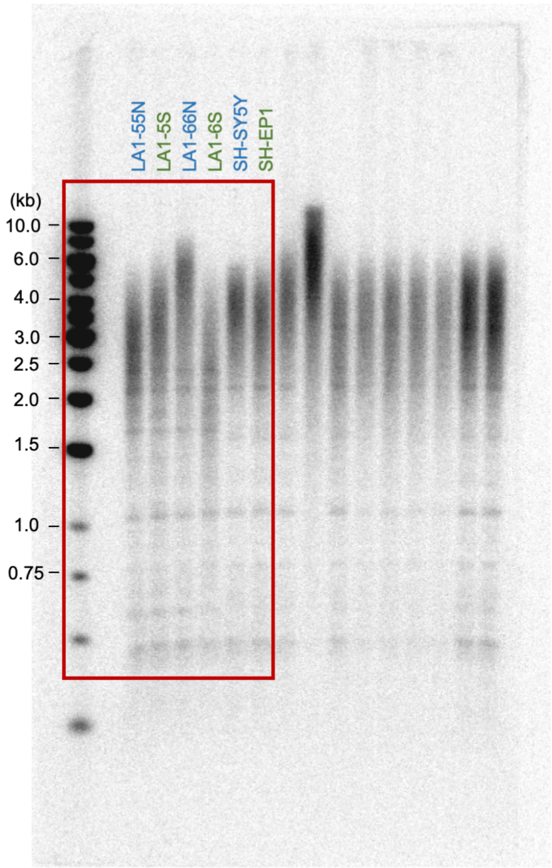

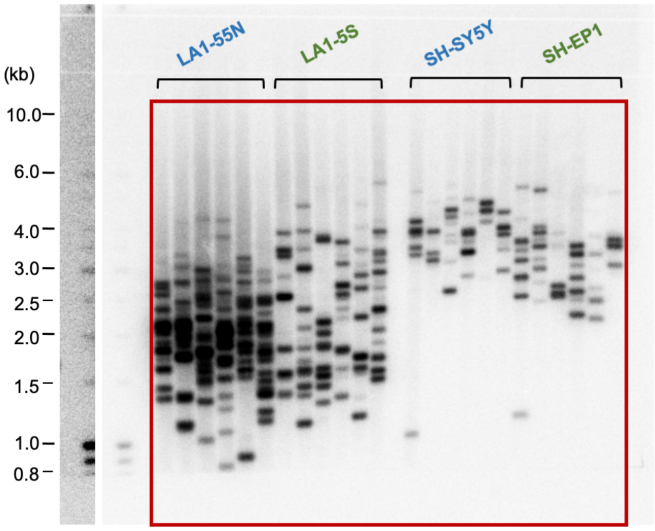

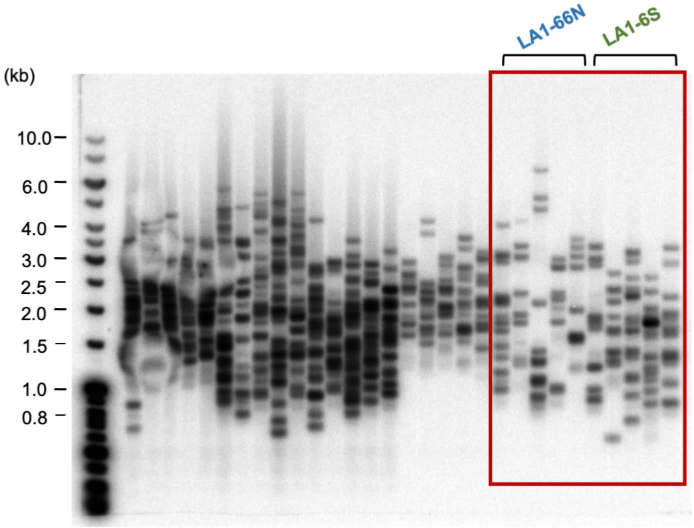

Original images for Fig. 2d and 2e

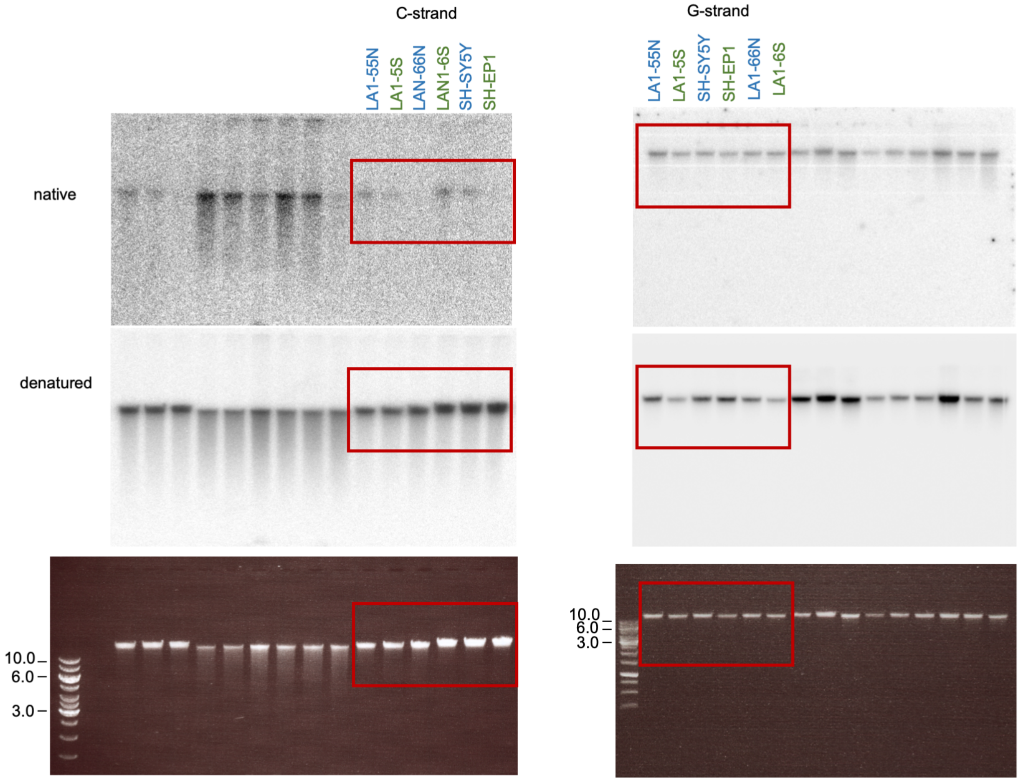

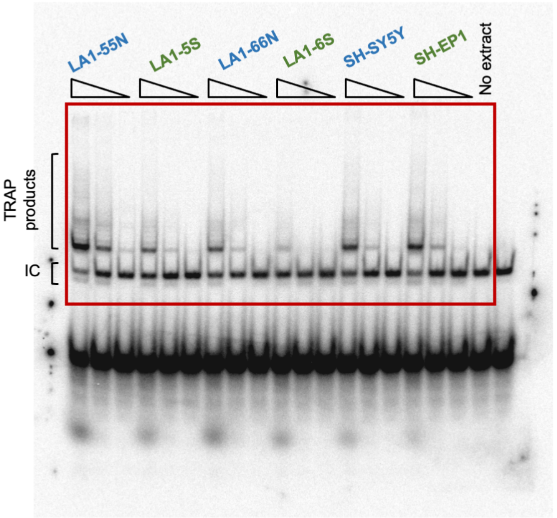

Original images for Fig. 3

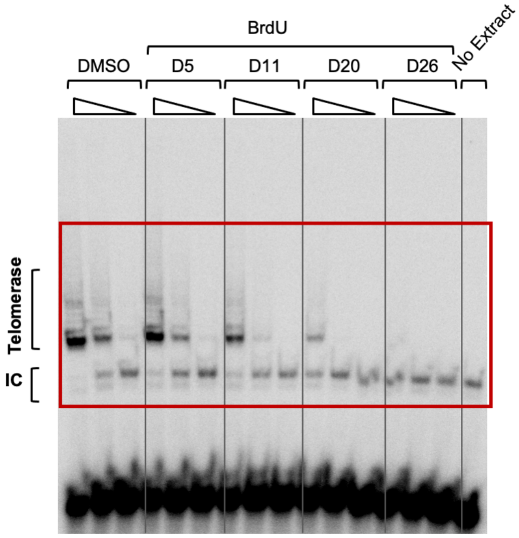

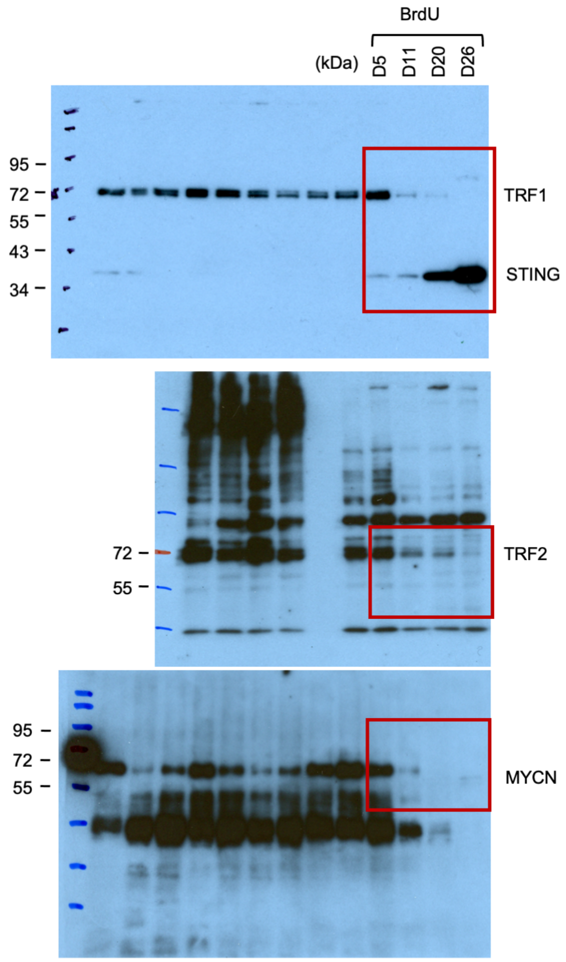

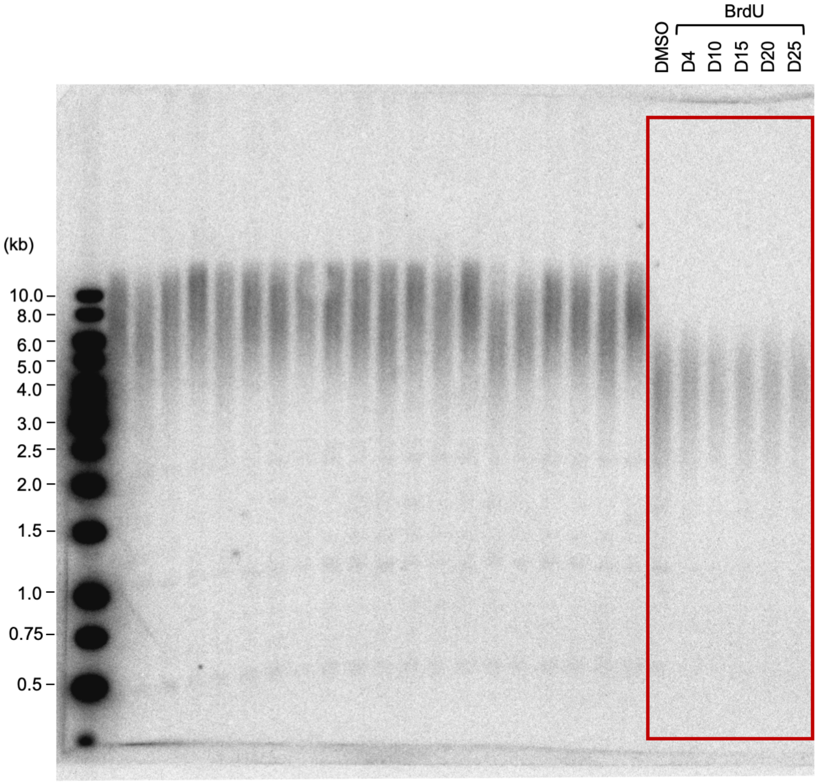

Original images for Fig. 4

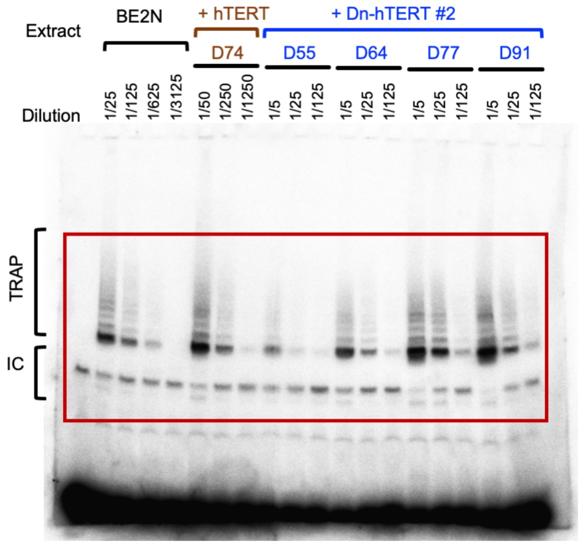

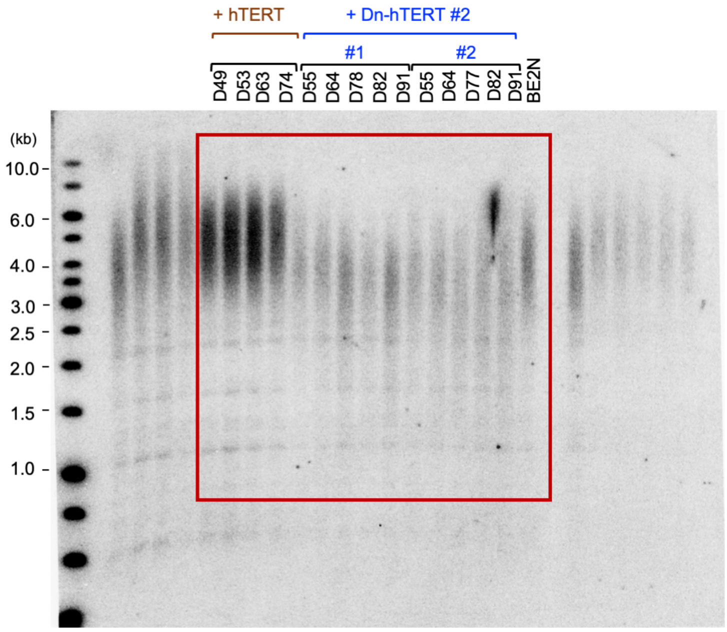

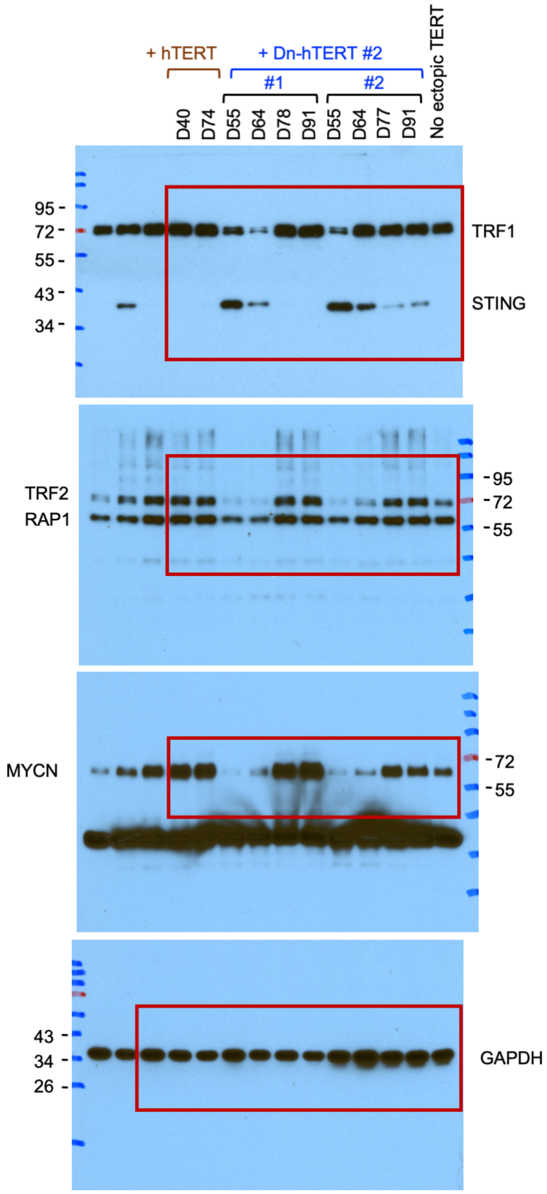

Original images for Fig. 5

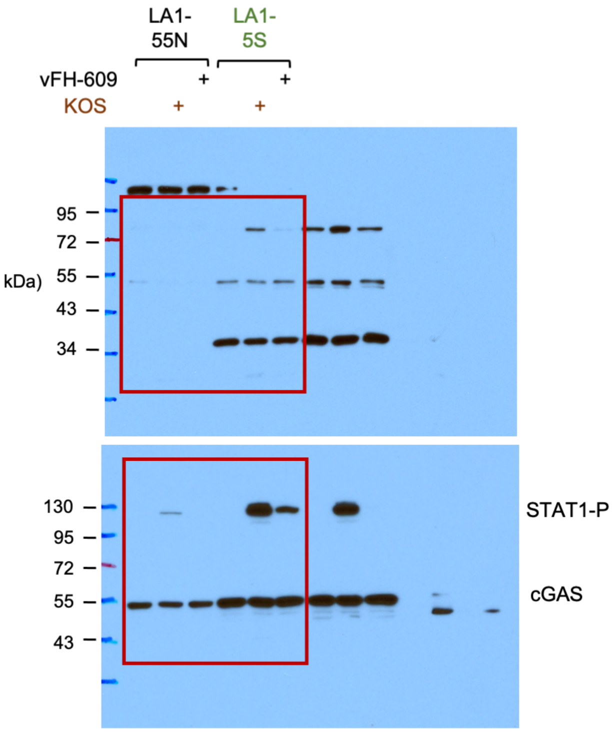

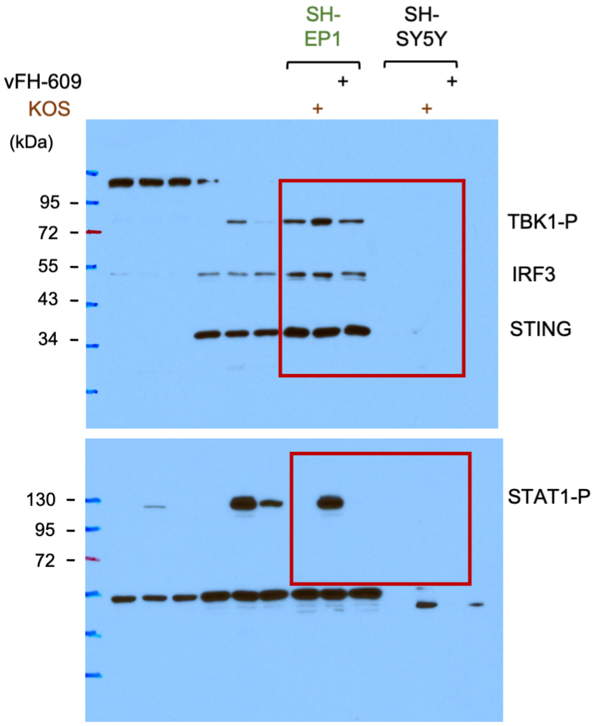

Supplement: Supplementary file 2 — Supplementary Information [file 42003_2021_2821_MOESM2_ESM.pdf]
