# Supplementary material for: Reciprocal impacts of telomerase activity and ADRN/MES differentiation state in neuroblastoma tumor biology
Source: Commun Biol. 2021 Nov 19;4:1315. doi: 10.1038/s42003-021-02821-8 (PMC8604896; doi:10.1038/s42003-021-02821-8)
Supplement: Supplementary file 5 — Reporting Summary [file 42003_2021_2821_MOESM5_ESM.pdf]

nature research

Corresponding author(s): Neal Lue
Last updated by author(s): Oct 10, 2021

Reporting Summary

Nature Research wishes to improve the reproducibility of the work that we publish. This form provides structure for consistency and transparency in reporting. For further information on Nature Research policies, see Authors & Referees and the Editorial Policy Checklist .

Statistics

For all statistical analyses, confirm that the following items are present in the figure legend, table legend, main text, or Methods section.

n/a	Confirmed	
[ ]	[x]	The exact sample size (*n*) for each experimental group/condition, given as a discrete number and unit of measurement
[ ]	[x]	A statement on whether measurements were taken from distinct samples or whether the same sample was measured repeatedly
[ ]	[x]	The statistical test(s) used AND whether they are one- or two-sided<br>*Only common tests should be described solely by name; describe more complex techniques in the Methods section.*
[ ]	[x]	A description of all covariates tested
[x]	[ ]	A description of any assumptions or corrections, such as tests of normality and adjustment for multiple comparisons
[ ]	[x]	A full description of the statistical parameters including central tendency (e.g. means) or other basic estimates (e.g. regression coefficient) AND variation (e.g. standard deviation) or associated estimates of uncertainty (e.g. confidence intervals)
[ ]	[x]	For null hypothesis testing, the test statistic (e.g. *F*, *t*, *r*) with confidence intervals, effect sizes, degrees of freedom and *P* value noted<br>*Give P values as exact values whenever suitable.*
[x]	[ ]	For Bayesian analysis, information on the choice of priors and Markov chain Monte Carlo settings
[x]	[ ]	For hierarchical and complex designs, identification of the appropriate level for tests and full reporting of outcomes
[x]	[ ]	Estimates of effect sizes (e.g. Cohen's *d*, Pearson's *r*), indicating how they were calculated

Our web collection on statistics for biologists contains articles on many of the points above.

Software and code

Policy information about availability of computer code

Data collection	ImageQuant TL 8.2 (GE Healthcare Inc.)
Data analysis	Microsoft Excel; Prism 9.0 (GraphPad Software Inc.); TeSLAQuant (Lai et al., Nat. Commun 8, 1356); R 4.0.4 (https://r-project.org)

For manuscripts utilizing custom algorithms or software that are central to the research but not yet described in published literature, software must be made available to editors/reviewers. We strongly encourage code deposition in a community repository (e.g. GitHub). See the Nature Research guidelines for submitting code & software for further information.

Data

Policy information about availability of data

All manuscripts must include a data availability statement. This statement should provide the following information, where applicable:

- Accession codes, unique identifiers, or web links for publicly available datasets
- A list of figures that have associated raw data
- A description of any restrictions on data availability

The datasets generated and/or analysed during the current study are included within the paper and Supplementary Information or available from the corresponding author upon reasonable request. The RNA-seq data have been deposited at GEO (accession GSE171404).

Field-specific reporting

Please select the one below that is the best fit for your research. If you are not sure, read the appropriate sections before making your selection.

[x] Life sciences [ ] Behavioural & social sciences [ ] Ecological, evolutionary & environmental sciences

Life sciences study design

All studies must disclose on these points even when the disclosure is negative.

Sample size	Multiple, independently derived cell lines were analyzed. Pharmacological and genetic manipulation of cell lines were performed with multiple, independent cell populations.
Data exclusions	Data were excluded when experiments failed or were of poor quality as to prevent clear interpretations.
Replication	Each experiment was repeated multiple times to ensure reproducibility.
Randomization	Not applicable
Blinding	Not customary for the type of experiments carried out in this study.

Reporting for specific materials, systems and methods

We require information from authors about some types of materials, experimental systems and methods used in many studies. Here, indicate whether each material, system or method listed is relevant to your study. If you are not sure if a list item applies to your research, read the appropriate section before selecting a response.

Materials & experimental systems

n/a	Involved in the study	
☐	☒	Antibodies
☐	☒	Eukaryotic cell lines
☒	☐	Palaeontology
☒	☐	Animals and other organisms
☒	☐	Human research participants
☒	☐	Clinical data

Methods

n/a	Involved in the study	
☒	☐	ChIP-seq
☒	☐	Flow cytometry
☒	☐	MRI-based neuroimaging

Antibodies

Antibodies used	mouse anti-TRF1 (SCBT, sc-56807), mouse anti-TRF2 (Novusbio, NB100-56506), mouse anti-RAP1 (SCBT, sc-53434), mouse anti-TIN2 (Novusbio, NB600-1522), rabbit anti-TPP1 (Bethyl, A303-069A-T), rabbit anti-POT1 (Novusbio, NB500-176), mouse anti-GAPDH (ABclonal, AC002), mouse anti-TERT (SCBT, sc-393013), mouse anti-STN1/OBFC1 (SCBT, sc-374178), mouse anti-PRIM1 (SCBT, sc-390265), mouse anti-POLA2 (SCBT, sc-398255), mouse anti-RPA70 (SCBT, sc-48425), rabbit anti-TZAP (Proteintech, 24665-1-AP), mouse anti-ATRX (SCBT, sc-55584), mouse anti-RTEL1 (SCBT, sc-515427), mouse anti-RIF1 (SCBT, sc-515573), mouse anti-MYCN (SCBT, sc-53993), mouse anti-IRF3 (SCBT, sc-33641), rabbit anti-STING (Cell Signaling, #13647), mouse anti-cGAS (SCBT, sc-515777), mouse anti-SLUG (SCBT, sc-166476), mouse anti-GATA3 (SCBT, sc-269), rabbit anti-phospho-STAT1 (Cell Signaling, #9167), rabbit anti-phospho-TBK1 (Cell Signaling, #5384).
Validation	All the antibodies were purchased from commercial sources with associated validations.

Eukaryotic cell lines

Policy information about cell lines

Cell line source(s)	NB cell lines were established at MSKCC or obtained from Robert Ross.
Authentication	BE(2)N, BE(2)C, and LAN-1 (the parent of LA1-55N, LA1-5S, LA1-66N and LA1-6S) were authenticated by STR analysis.
Mycoplasma contamination	The cell lines were not tested for mycoplasma contamination.
Commonly misidentified lines (See ICLAC register)	N/A
